# Retraction: Inhibitor 9 Combined With Androgen Deprivation Therapy or Chemotherapy Delays the Malignant Behavior of Castration-Resistant Prostate Cancer Through K-Ras/PLCϵ/PKCϵ Signaling Pathway

**DOI:** 10.3389/fonc.2021.707608

**Published:** 2021-05-31

**Authors:** Frontiers Editorial Office

**Affiliations:** Frontiers Media SA, Lausanne, Switzerland

**Keywords:** KRAS mutation G12C, inhibitor 9, castration-resistant prostate cancer, drug resistance, metastasis

Please find the retraction statement below:

Following publication, concerns were raised regarding image manipulation and duplication. The authors failed to provide a satisfactory explanation during the investigation, which was conducted in accordance with Frontiers’ policies.

This retraction was approved by the Chief Editors of Frontiers in Oncology and Chief Executive Editor. The authors agreed to this retraction.

